# Ferroptosis in Lung Cancer: From Molecular Mechanisms to Prognostic and Therapeutic Opportunities

**DOI:** 10.3389/fonc.2021.792827

**Published:** 2021-12-02

**Authors:** Peyman Tabnak, Zanyar HajiEsmailPoor, Soroush Soraneh

**Affiliations:** ^1^ Faculty of Medicine, Tabriz University of Medical Sciences, Tabriz, Iran; ^2^ Faculty of Medicine, Urmia University of Medical Sciences, Urmia, Iran

**Keywords:** lung cancer, ferroptosis, biomarkers, cell death, Nrf2, iron metabolism, ROS, immunity

## Abstract

Lung cancer is the second commonly diagnosed malignancy worldwide and has the highest mortality rate among all cancers. Tremendous efforts have been made to develop novel strategies against lung cancer; however, the overall survival of patients still is low. Uncovering underlying molecular mechanisms of this disease can open up new horizons for its treatment. Ferroptosis is a newly discovered type of programmed cell death that, in an iron-dependent manner, peroxidizes unsaturated phospholipids and results in the accumulation of radical oxygen species. Subsequent oxidative damage caused by ferroptosis contributes to cell death in tumor cells. Therefore, understanding its molecular mechanisms in lung cancer appears as a promising strategy to induce ferroptosis selectively. According to evidence published up to now, significant numbers of research have been done to identify ferroptosis regulators in lung cancer. Therefore, this review aims to provide a comprehensive standpoint of molecular mechanisms of ferroptosis in lung cancer and address these molecules’ prognostic and therapeutic values, hoping that the road for future studies in this field will be paved more efficiently.

## Highlights

- GPX4, system $X_{c}^{-}$ , NRF2, p53, and UPS are the main ferroptosis regulators in lung cancer.
- Ferroptosis has a close relationship with the immune system status in lung cancer.
- The expression of ferroptosis-related genes including, ALOX15, PEBP1, GLS2, and PHKG2, positively predict prognosis.
- The expression of ferroptosis-related genes, including CISD1, ACSL3, FANCD2, and SLC7A11, negatively predict prognosis.
- Combining ferroptosis inhibitors with radiotherapy or chemotherapy synergistically kills lung tumors.

## 1 Introduction

With 11.4% and 18% of prevalence and total cancer death rate, respectively, lung cancer is the second common cancer and the leading cause of cancer death worldwide (1). Though tobacco smoking still is the critical risk factor for lung cancer, the importance of non-tobacco risk factors such as environmental and occupational exposures, chronic lung disease, and lifestyle factors is growing (2). Based on cell origin, lung cancers are divided into two primary subtypes, including non-small-cell lung carcinoma (NSCLC, more common form) and small-cell lung carcinoma (SCLC, less common form). Lung adenocarcinoma (LUAD), as the most common histological subtype of NSCLC, accounts for 38.5% of all lung cancer cases (3). Currently, conventional treatment choices include surgery and adjuvant therapies (e.g., chemotherapy, radiotherapy, and targeted therapy); however, half of the patients succumb within the first year of diagnosis, and five years overall survival is below 18 percent (4). Given the facts mentioned above, the health burden caused by lung cancer is remarkable and extensive efforts have been made to improve the disease in recent years. Understanding the molecular mechanism of lung cancer opens up new horizons for developing novel strategies to manage and fight against this malignancy (5). Ferroptosis, discovered in 2012, was first identified as a type of oxidative iron-dependent programmed cell death (PCD) different from apoptosis, necrosis, and autophagy. Following treatments with small molecules such as erastin, some morphological changes like chromatin condensation, cytoplasmic and organelle swelling, formation of double-membrane vesicles, shrunken mitochondria, and plasma membrane rupture were observed in affected cells (6). This process initiates with the accumulation of various pro-ferroptotic molecules contributing to lipid peroxidation through the production of reactive oxygen species (ROS) under the assistance of iron (7). Our understanding of ferroptosis has been growing over the last decade, and the numbers of ferroptosis regulators are increasing (8). Two main inhibitors of ferroptosis, including system $X_{c}^{-}$ and glutathione peroxidase 4 (GPX4), prevent phospholipid peroxidation under physiological conditions. Control of cellular metabolism by different nutrients, intra/intercellular signaling pathways (e.g., p53 and NRF2), and environmental stress play an essential role in the synthesis of ferroptosis stimulators such as ROS and phospholipids containing polyunsaturated fatty acid chains (PUFA-PLs) (9). Ferroptosis is effective in eliciting a therapeutic response by experimental reagents (e.g., erastin and RSL3), approved drugs (e.g., sulfasalazine and artemisinin), ionizing radiation, and cytokines (e.g., IFNγ and TGFβ1), leading to inhibition of tumor growth in various cancer types (10). Therefore, cell death caused by ferroptosis is a great step toward cancer therapy. A study has shown that in Xuanwei area of China which the incidence of lung cancer is very high, ferroptosis dysregulation may implicate in the development of the disease (11). In this study, we particularly aimed to review the role of ferroptosis in lung cancer and provide insights into molecular mechanisms, prognostic and therapeutic importance of ferroptosis regulators.

## 2 Ferroptosis Regulators in Lung Cancer

### 2.1 Ferroptosis Suppressors

#### 2.1.1 System $X_{c}^{-}$

**Figure 1:**
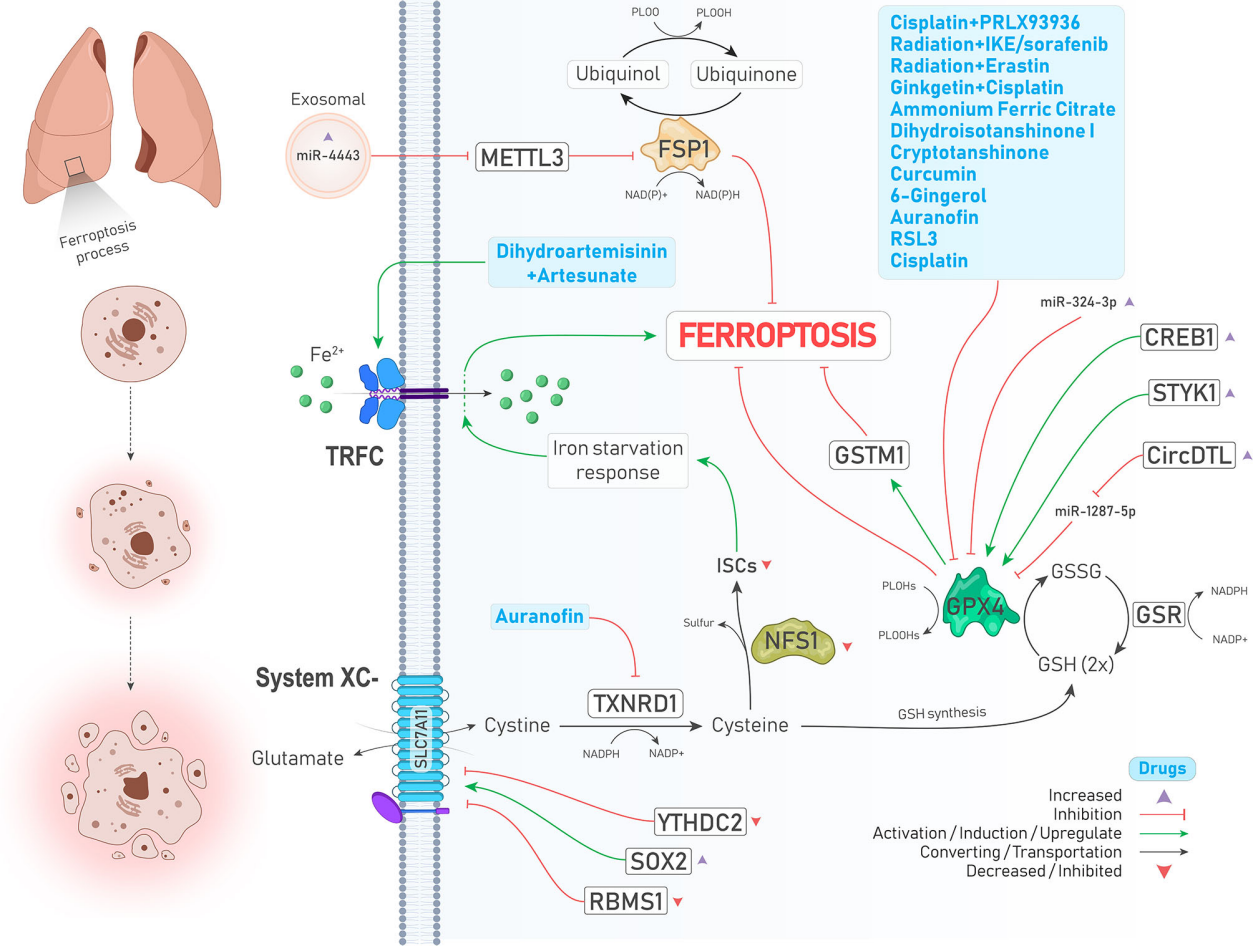
An illustration showing regulation of ferroptosis suppressors, including system $X_{c}^{-}$ ,GPX4, FSP1, and NFS1 in lung cancer by different molecules and treatments. System $X_{c}^{-}$ is upregulated and downregulated due to increase of SOX2 (↓ Ferroptosis) and decrease of YTHDC2 levels (↑ Ferroptosis), respectively. TXNRD1 is inhibited by auranofin (↑ Ferroptosis). Inhibited NFS1 and subsequently decreased ISCs biosynthesis leads to an iron starvation response which causes Fe^2+^ influx to cells by TRFC (↑ Ferroptosis). As a ferroptosis inhibitor, GPX4 is suppressed by a group of drugs depicted in the blue box (↑ Ferroptosis). In addition, GPX4 is positively upregulated by enhanced expressions of CREB and STYK1 (↓ Ferroptosis). FSP1, which inhibits ferroptosis independently, is upregulated upon increased expression of oncogenic miR-4443 (↑ Ferroptosis).

System $X_{c}^{-}$ (xCT) is a cystine/glutamate antiporter consisting of two subunits, including SLC3A2 and SLC7A11, which is responsible for exporting glutamate and importing cystine (12). Upon this transportation, cystine in the cytosol following an NADPH-consuming reduction reaction by thioredoxin reductase 1 (TXNRD1) is converted to cysteine, an antioxidant amino acid and precursor of tripeptide glutathione (GSH). Moreover, GSH is also a potent antioxidant that is used by glutathione peroxidase 4 (GPX4) for inhibition of ferroptosis by preventing the accumulation of lipid hydroperoxides (LOOHs) and converting them to lipid alcohols (LOHs) (**Figure 1**). Therefore, system $X_{c}^{-}$ as a membrane heterodimer is implicated in inhibition of ferroptosis under oxidative stress to provide cancer cell survival (13). Up to now, various studies have pointed to the role of this system in the regulation of ferroptosis in *lung cancer*, most of them representing SLC7A11 as the targets of various novel drugs and upstream regulators (**Figure 1**). For example, a recent study has elucidated that there is a positive relationship between the level of transcription factor SOX2 and SLC7A11 in lung cancer stem-like cells (CSLC), and upregulation of SOX2 in lung tumors can highly inhibit ferroptosis and increase resistance to imidazole ketone erastin (IKE), a strong inducer of ferroptosis (14). Likewise, a similar relationship was observed between RNA binding protein RBMS1 and SLC7A11, in a way that depletion of RBMS1 sensitizes lung cancer cells to ferroptosis and radiotherapy (15). Moreover, Ma et al. have reported that YT521-B homology domain containing 2 (YTHDC2) is a downregulated tumor suppressor in lung adenocarcinoma (LUAD), which can suppress the activity of system $X_{c}^{-}$ through targeting mRNA encoding SLC7A11 (16). However, their further investigations revealed that inhibition of SLC7A11 by YTHDC2 is not enough to induce ferroptosis in lung adenocarcinoma. Therefore, they proposed that SLC3A2 as another subunit of system $X_{c}^{-}$ should be inhibited by YTHDC2 as well. High-level induction of YTHDC2 could inhibit the expression of SLC3A2 indirectly by inhibiting the expression of HOXA13. Further *in vivo* experiments also confirmed that the induction of YTHDC2 can highly contribute to ferroptosis. Therefore, elevating the levels of YTHDC2 was shown to be a promising strategy in LUAD to induce ferroptosis by selectively inhibiting the expression of both subunits of system $X_{c}^{-}$ in tumor cells (17).

#### 2.1.2 GPX4

GPX4, a selenoperoxidase and a key upstream regulator of ferroptosis, plays two roles at the same time including, converting GSH to glutathione disulfide (GSSG) and reducing phospholipid hydroperoxides (PLOOHs) to their corresponding alcohol (PLOHs), thereby preventing the accumulation of lipid peroxides and leading to suppression of ferroptosis. In addition, GSSG can be recycled to GSH under the action of glutathione–disulfide reductase (GSR) using the electrons provided by NADPH/H^+^ (9) (**Figure 1**). Various studies have reported that GPX4 is upregulated in different cancers, including LUAD, and is associated with poor prognosis of patients and chemotherapy resistance. Suppression of GPX4 by a small molecule inhibitor named RSL3 strongly enhances the anticancer effects of cisplatin *in vivo*. In other words, RSL3 combined with cisplatin could induce ferritinophagy/ferroptosis (18). Another study has also shown that the levels of GPX4 and mTORC1 are higher in NSCLC cells, and inhibition of these molecules provides a promising strategy to overcome lapatinib resistance *in vivo* (19). cAMP response element-binding protein (CREB) is also an upregulated oncogene in LUAD tissues that positively regulates the expression of GPX4, and their levels are closely related to tumor size and stage (20). A similar relationship was also observed between serine threonine tyrosine kinase 1 (STYK1) and GPX4 in NSCLC. In brain metastasis of lung adenocarcinoma, Glutathione S‐transferase M1 (GSTM1) is another protein that is stablized by GPX4 and concurrently result in inhibition of ferroptosis and subsequent resistance to platinum through increasing GSH consumption (21). We will further show that many drugs and upstream signaling pathways significantly affect the regulation of GPX4 in lung cancer (22)(**Figure 1** and **Table 2**).

#### 2.1.3 FSP1

Recent studies have identified that GPX4 is not the only ferroptosis suppressor in human cancer cells, and ferroptosis suppressor protein 1 (FSP1, formerly known as AIFM2) independent of GSH suppresses lipid peroxidation and subsequent ferroptosis through converting ubiquinone (coenzyme Q10, CoQ10) to ubiquinol (CoQH2, reduced form of CoQ10) using an NADPH-consuming reduction reaction (**Figure 1**). Therefore, this pathway is also referred to as the CoQ-dependent pathway (9). Bersuker et al. showed that FSP1 expression positively correlates with ferroptosis resistance in various cell lines, particularly in the xenograft mouse model of *lung cancer (* 23).

#### 2.1.4 NFS1

Cysteine desulfurase nitrogen fixation 1 homolog (NFS1) is an enzyme that extracts sulfur from cysteine for the biosynthesis of iron-sulfur clusters (ISCs) and is expressed at higher levels in differentiated lung adenocarcinomas. A study has demonstrated that suppressing NFS1 as well as ISCs biosynthesis triggers an iron-starvation response and subsequent iron influx to cells by molecules such as transferrin receptor protein (TRFC), thereby promoting ferroptosis and inhibiting tumor growth in lung tumors (24, 25) (**Figure 1**).

#### 2.1.5 NRF2

**Figure 2:**
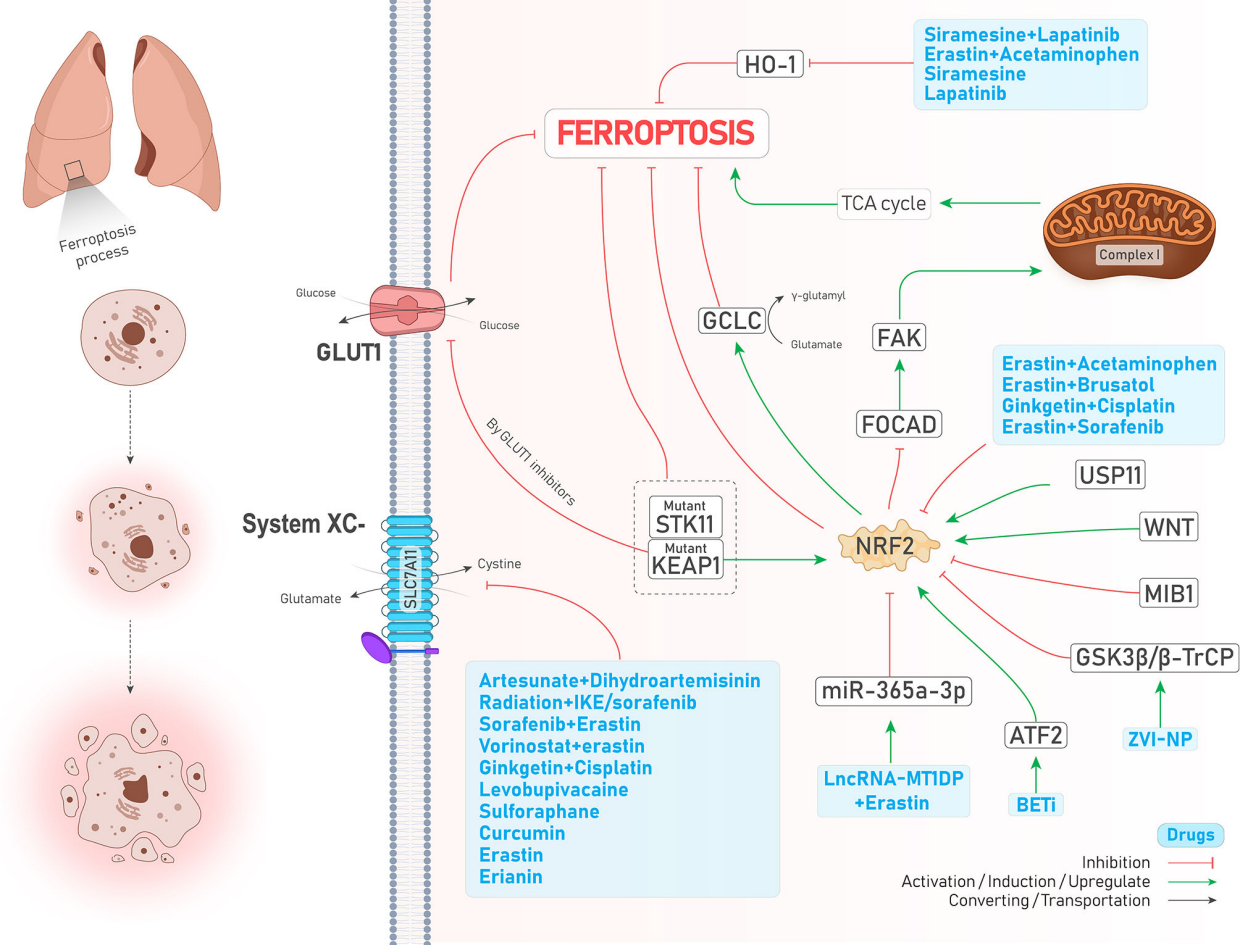
An illustration showing the role of NRF2 in regulating ferroptosis in lung cancer and its control by different molecules and treatments. NRF2 inhibits ferroptosis. KEAP1-mutant cells overexpress NRF2 and inhibit ferroptosis; while using GLUT1 inhibitor agents in these cells induces ferroptosis. Co-mutant STK11 and KEAP1 cells (depicted by dotted box) are more resistant to ferroptosis (↓ Ferroptosis) due to increased levels of GPX4 and SLC7A11 (which is not shown here). The combination of lncRNA-MT1DP and erastin inhibits NRF2 by increasing the activity of miR-365a-3p (↑ Ferroptosis). ATF2 activation by BET inhibitors causes NRF2 upregulation (↓ Ferroptosis). ZVI-NP mediates NRF2 degradation by inducing GSK3β/β-TrCP (↑ Ferroptosis). USP11 activation causes NRF2 induction (↓ Ferroptosis). A significant number of drugs (depicted in the blue box) inhibits NRF2 (↑ Ferroptosis). FOCAD-FAD axis, which mediates ferroptosis by induction of TCA cycle and complex I in the mitochondria, is inhibited by NRF2 (↓ Ferroptosis). Higher levels of NRF2 increase the activity of GCLC (↓ Ferroptosis). A large group of drugs (the biggest blue box) could inhibit system $X_{c}^{-}$ (↑ Ferroptosis). HO-1 naturally causes ferroptosis (not shown here); while its degradation by another group of drugs (depicted in the blue box) inhibits ferroptosis (↓ Ferroptosis).

Transcription factor nuclear factor erythroid 2-related factor 2 (NFE2L2 or NRF2) is an overriding antioxidant transcription regulator of genes involved in lipid peroxidation and the accumulation of free iron (26). Takahashi et al. emphasized that hyperactivation of NRF2 is required for proliferation and survival of 3D culture models of lung tumors through preventing ferroptosis. However, NRF2 downregulation was not enough to suppress ferroptosis since GPX4 levels were increased, proposing that enhanced oxidative stress caused by NRF2 downregulation might activate other cytoprotective signaling pathways, including nuclear factor-κB (NF-κB), involved in activating downstream antioxidant enzymes. Notably, simultaneous inhibition of NRF2 and GPX4 could efficiently induce ferroptosis (27). The study by Liu et al. showed that Wnt signaling pathway through provoking NRF2 increases the activity of GPX4 in brain metastasis of lung adenocarcinoma cells, thereby resulting in chemoresistance to platinum (21). In addition, the activity of NRF2 positively reflects erastin resistance in isogenic lung cancer cell models, regardless of Kelch-like ECH-associated protein 1(KEAP1) mutation (28), a molecule that represses NRF2 *via* its ubiquitin proteasomal degradation in normal conditions (29). Likewise, KEAP1-mutant lung cancer cells were shown to have higher levels of NRF2 and its downstream target SLC7A11. In addition, KEAP1 deficient lung tumors were shown to be sensitive to inhibition of glucose transporter 1 (GLUT1) due to their glucose dependency (30). Similarly, Wohlhieter et al. (31) showed that LUAD tumors with concurrent mutations in serine/threonine kinase 11 (STK11) and KEAP1 were more resistant to ferroptosis since the activity of NRF2 pathway and targets involved in ferroptosis such as SLC7A11 and GPX4 were increased, leading to worse overall survival and enhanced tumor proliferation both *in vivo* and *in vitro* (**Figure 2**). Moreover, their further investigations turned out that stearoyl-CoA desaturase (SCD1, SCD) was necessary for the proliferation of the cells mentioned above (**Figure 2**). SCD1 was previously shown to participate in aberrant lipid metabolism and promoted cell growth in lung cancer (32); therefore, its genetic and pharmacological inhibition could sensitize STK11/KEAP1 co-mutant cells to ferroptosis induction even *in vivo*. Noteworthy to mention, the role of mitochondria and the tricarboxylic acid (TCA) cycle has been highlighted recently in regulating ferroptosis (33). NRF2 can also affect mitochondrial function. For example, in NSCLC, FOCAD-FAK signaling was shown to be involved in cysteine deprivation-induced ferroptosis, and NRF2 was shown to inhibit the FOCAD-FAK signaling axis and induce ferroptosis *via* increasing the activity of Complex I in the mitochondrial electron transport chain (ETC) and TCA cycle. However, inhibiting NRF2 is not enough for ferroptosis induction since the FOCAD-FAK axis does not affect GPX4. Therefore, adding an NRF2 inhibitor such as brusatol concurrent with erastin can promote ferroptosis induction better in NSCLC cells (34) (**Figure 2**). Similarly, a novel study conducted by Kang et al. has highlighted the role of the NRF2 signaling pathway in cystine depletion conditions. NSCLC cells with higher expression levels of NRF2 produce many γ-glutamyl-peptides due to increased activity of glutamate-cysteine ligase catalytic subunit (GCLC). Following production of γ-glutamyl-peptide, glutamate is not accumulated in the cytosol anymore and this process leads to ferroptosis inhibition (35). Activating transcription factor 2 (ATF2) is another protein with an oncogenic role in lung cancer and increases NRF2 expression following treatments with a group of drugs named BET inhibitors (BETi, with the ability to induce ferroptosis in breast cancer) in LUAD, leading to ferroptosis resistance (36). In addition, a very recent study has shown that the expression of E3 ubiquitin ligase Mindbomb 1 (MIB1) is upregulated in a group of lung squamous and adenocarcinoma cells and correlates negatively with patients’ survival. Nevertheless, cells overexpressing MIB1 are more sensitive to ferroptosis due to proteasomal degradation of NRF2 by MIB1 (37). Taken together, due to the prominent role of NRF2 in regulating ferroptosis, targeting it by various treatments can significantly induce ferroptosis (see **Table 2**).

### 2.2 Ferroptosis Inducer

#### 2.2.1 ACSL4

Acyl-CoA synthetase long-chain family member 4 (ACSL4) is a crucial enzyme responsible for lipid metabolism, which converts PUFAs to PUFA-CoAs, and following the action of LPCAT3, the products of this reaction are esterified into phospholipid containing polyunsaturated fatty acid chain (PUFA-PLs). Then, these PUFA-PLs are oxidized by another enzyme named ALOX15 into PL-PUFA-OOHs. Since PL-PUFA-OOHs can trigger ferroptotic cell death, the activity of the abovementioned enzymes contributes to the promotion of ferroptosis (38). Surprisingly, although ALOX15 and ACSL4 facilitate the ferroptosis process, recent studies showed that higher expression of these molecules was associated with increased cancer malignant features. Thereby, these molecules can act as double-edged swords either by promoting or inhibiting cancer progression (39). However in SCLC, Bebber et al. showed that ACSL4 and LPCAT3 are expressed at higher levels in non-neuroendocrine (non-NE-SCLC) than NE-SCLC, contributing to ferroptosis sensitivity and resistance, respectively in the abovementioned cells. Moreover, they proposed that TRX antioxidant pathway is overactivated in NE-SCLC cells, and its inhibition by auranofin alongside treatment with buthionine sulfoximine (BSO), as a GSH level reducer, could successfully induce ferroptosis and inhibit tumor progression in mice with xenograft NE-SCLC tumors (40). In addition, ACSL4 was found to act as a tumor suppressor and a favorable prognostic factor in patients with LUAD and promoted ferroptosis but inhibited tumor cell survival, invasion, and migration. Most interestingly, a high-fat diet could reverse these effects *via* downregulating ACSL4 both *in vivo* and *in vitro (* 41).

### 2.3 Signaling Pathways and Their Crosstalk With Ferroptosis in Lung Cancer

#### 2.3.1 EGFR and MAPK

**Figure 3:**
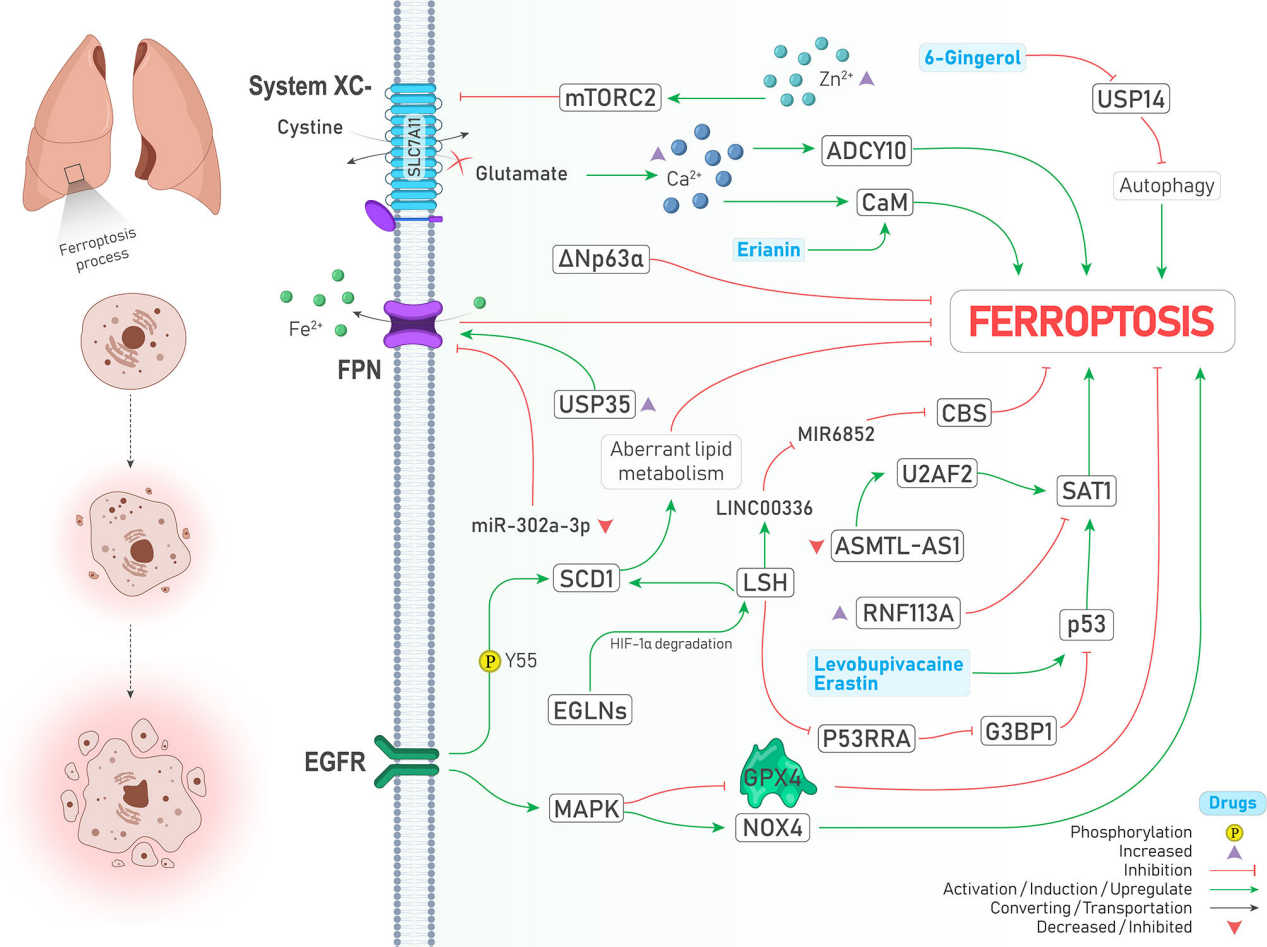
An illustration showing signaling pathways involved in regulating ferroptosis in lung cancer and their possible crosstalk together. MAPK activation leads to GPX4 inhibition and NOX4 activation (↑ Ferroptosis). SCD1 stabilization by EGFR *via* its Y55 phosphorylation inhibits ferroptosis through aberrant lipid metabolism (↓ Ferroptosis). Under the hypoxic condition, EGLNs degrades HIF-1α. Therefore, LSH expression is increased and induces SCD1 expression (↓ Ferroptosis). LSH can upregulate lncRNA-LINC00336. This interaction results in CBS inhibition *via* sponging/inhibiting MIR6852 (↓ Ferroptosis). In addition, LSH downregulates lncRNA-NEAT1 and inhibits p53 *via* G3BP1 downregulation (↓ Ferroptosis). Upregulation of SAT1 by p53 promotes ferroptosis, and RNF113A deficiency causes ferroptosis by increasing SAT1. Two drugs induce the expression of p53 and drive the cell toward ferroptosis (↑ Ferroptosis). ΔNp63α inhibits ferroptosis. USP35 overexpression leads to ferroptosis suppression by stabilization of FPN (↑ Ferroptosis). Moreover, FPN is inhibited upon decreased expression of miR-302a-3p (↑ Ferroptosis). Zinc intoxication causes ferroptosis by inhibition of the System $X_{c}^{-}$ inhibition circumstances, glutamate is accumulated in the cell, which results in the calcium ions accumulation. This accumulation induces ferroptosis by CaM and ADCY10 activation (↑ Ferroptosis). Erianin activates CaM (↑ Ferroptosis). USP14 inhibition by 6-Gingerol leads to autophagy induced ferroptosis (↑ Ferroptosis).

The epidermal growth factor receptor (EGFR) pathway is involved in the progression of various cancers, and its mutation is frequently seen in lung adenocarcinomas (42). It is noteworthy to mention that the previously mentioned SCD1 is stabilized by EGFR *via* Y55 phosphorylation and contributes to cancer progression in lung cancer (32). Moreover, activation of mitogen-activated protein kinase (MAPK) as a downstream target of the EGFR pathway is required to induce ferroptosis (**Figure 3**). Following cystine-deprived conditions, those NSCLC cells with the highest MAPK signaling activity significantly lose their viability through a ferroptosis process caused by inhibited and promoted expression of GPX4 and NOX4, respectively (43).

#### 2.3.2 HIF-1α

Hypoxia, as a hallmark of cancer, leads to excess tumor vascularization and progression (44). Activation of both MAPK and EGFR pathways can induce the expression of hypoxia-inducible factor 1-alpha (HIF-1α) (44, 45). In addition, induction of HIF-1 by EGFR can make A549 lung cancer cells resistant to another type of PCD called anoikis under the lipid raft-disrupting stress (45). Emerging evidence also supports the role of HIFs in the induction of ferroptosis (46, 47). Jiang et al. showed that iron-dependent enzymes Egl nine homolog (EGLNs) under hypoxia conditions degrades HIF-1α (**Figure 3**), leading to increased lymphoid-specific helicase (LSH) expression, a chromatin remodeling factor that acts as an oncogene and ferroptosis inhibitor in lung cancer *via* increasing the expression of SCD1 (48, 49).

#### 2.3.3 P53

Tumor suppressor P53 has a dual role in regulating ferroptosis. Spermidine/spermine N1-acetyltransferase (SAT1) is one of those proteins which is upregulated by p53 and responsible for oxidative stress and ferroptosis (50, 51). However, SAT1 is under the control of factors other than p53. For instance, RNF113A is an oncogene RNA-binding protein whose deficiency provokes ferroptosis *via* promoting SAT1 expression (**Figure 3**) and was upregulated and contributed to cisplatin resistance in lung adenocarcinomas (52). Recently, it has been discovered that ΔNp63α as a major isoform of p63 can inhibit ferroptosis and oxidative stress independent of p53 and NRF2 activity in lung cancer through transcriptional controlling the expression of genes involved in glutathione synthesis (53).

#### 2.3.4 Proteasomal Degradation Pathway

Ubiquitin–proteasome system (UPS) belongs to a degradation pathway that controls lipid peroxidation and iron accumulation *via* degradation of molecules involved in ferroptosis (8). A recent review has also highlighted the role of ubiquitination in ferroptosis (54). Ubiquitin‐specific protease 35 (USP35) is a deubiquitinase that is overexpressed in lung cancer, and its knockdown, in addition to promoting ferroptosis and chemotherapeutic sensitivity to cisplatin and paclitaxel, inhibits lung cancer cell growth, colony formation, and tumor progression. The mechanism by which USP35 overexpression led to ferroptosis was attributed to ferroportin (FPN) stabilization, a protein responsible for exporting iron to the outside of the cells (**Figure 3**) **(** 55). In the same way, a similar relationship between deubiquitinase USP11 and NRF2 was found in patients with NSCLC (SCC subtype), leading to ferroptosis resistance and cell proliferation (**Figure 3**) **(** 56). Noteworthy to mention, the effects of some drugs in inducing ferroptosis are also carried out by the proteasome degradation system. For instance, concurrent treatment with siramesine alongside lapatinib was shown to induce ferroptosis *via* proteasome degradation of heme oxygenase-1 (HO-1) (57).

#### 2.3.5 Autophagy

Autophagy is another intracellular degradation pathway in which various molecules and organelles in the cell are engulfed and then degraded following the formation of lysosomal structures (8). There is close crosstalk between ferroptosis and autophagy, in a way that some of the recent reviews consider ferroptosis as a type of autophagy-dependent cell death (58, 59). The reason behind this assumption is that in the presence of some ferroptosis inducers such as erastin and RSL3, the formation of autophagosomes and components of the autophagy system is also provoked, and thereby ferroptosis is promoted (58). Some pieces of evidence support this relationship in lung cancer. Following inhibition of USP14 by 6-Gingerol (a natural product) in A549 lung cancer cells, the levels of ROS, iron concentration, and autophagosomes started to increase and contributed to increased expression of ferroptosis and autophagy-related proteins *in vivo* and *in vitro* (**Figure 3**). Therefore, it can be concluded that USP proteins are a common executer among autophagy and ferroptosis process (60). Similarly, treatment with curcumin, a well-known natural derived product, could significantly induce cell death both *in vivo* and *in vitro* through an autophagy-dependent ferroptosis mechanism and inhibition of autophagy reversed ferroptosis caused by curcumin in NSCLC (61).

#### 2.3.6 Hippo/YAP Pathway

Hippo is an evolutionarily conserved signaling pathway with potent tumor suppressor activities involved in determining cell fate. Its dysregulation highly induces cancer progression and therapy resistance through aberrant activation of two transcriptional coactivators, including yes-associated protein (YAP) and transcriptional coactivator with PDZ-binding motif (TAZ). When hippo signaling is off, YAP and TAZ are translocated to the nucleus and interact with DNA binding transcription factors named TEA domain family member (TEAD) to provoke the expression of genes involved in cell proliferation (62). Recently the role of Hippo/YAP/TAZ axis in regulating ferroptosis was described as dependent on the distance between cells and their contact. In other words, when there is no contact between the cells (low-density conditions), due to the inactivation of Hippo signaling, YAP and TAZ trigger the expression of genes responsible for ferroptosis induction rather than apoptosis. While this process is reversed when cells get closer to each other and extracellular E-cadherin stimulates Hippo pathway activation, leading to increased apoptosis (62). In LUAD, Zhang et al. demonstrated that the magnitude of YAP suppression is a crucial determiner of ferroptosis sensitivity. While inhibiting system $X_{c}^{-}$ by sorafenib could induce ferroptosis and decrease YAP levels, suppressing GPX4 could not, proposing that subsequent glutamate accumulation in the cytosol might sensitize cells to ferroptosis. Their further experiments showed that glutamate repletion conditions (e.g., system $X_{c}^{-}$ inhibition) provokes Ca^2+^ ions accumulation in the cytosol and ADCY10 initiates a cascade of reactions leading to YAP destabilization and subsequent ferroptosis (**Figure 3**). Since ADCY10 is expressed higher in advanced stage and therapy resistance LUAD cells, targeting ADCY10 as a molecule linked with YAP and ferroptosis was shown to have clinical significance (63).

#### 2.3.7 Calcium and Zinc Effects

The latest review on ferroptosis insists that ions other than iron (e.g., zinc) cannot induce ferroptosis (8); however, other ions can indirectly affect ferroptosis. As just mentioned earlier, at least in lung cancer, accumulation of Ca^2+^ subsequently triggers the reactions that lead to ferroptosis sensitivity (63). Likewise, treating lung cancer cells with erianin (a natural product) promoted ferroptosis *via* calcium/calmodulin (CaM) signaling activation, a pathway involved in increasing intracellular Ca^2+^ levels (**Figure 3**) **(** 64). Moreover, zinc intoxication in NSCLC cells upregulates the mTORC2/RICTOR pathway, resulting in system $X_{c}^{-}$ phosphorylation and subsequent ferroptosis (**Figure 3**). However, it should be noted that these effects of zinc are reversed after a specific time and are limited using iron chelator deferoxamine and vitamin E (65).

#### 2.3.8 mTOR Pathway

The mechanistic target of rapamycin (mTOR) is an essential negative regulator of autophagy (8). Moreover, GPX4 is also involved in regulating ferroptosis, and the levels of mTOR are positively correlated with GPX4 levels; therefore, mTOR inhibitors can induce autophagy-dependent ferroptosis (66). For this reason, “inhibition of GPX4 or mTORC1 overcomes resistance to Lapatinib *via* promoting ferroptosis in NSCLC cells” (19), while zinc intoxication increases mTORC2 activity and ferroptosis (65). This proposes the theory that mTORC1 and mTORC2 may have distinct impacts on regulating ferroptosis, at least in lung cancer. Moreover, it has been proposed that lung tumors, which in them NRF2 signaling pathway is activated more than usual, are more dependent on the mTOR pathway, and synergistic cooperation between NRF2 and mTOR signaling can enhance cell proliferation in 3D cultures (27).

### 2.4 Non-Coding RNAs

Non-coding-RNAs (ncRNAs) make up a considerable part of the human transcriptome. They are the hot topic of interest these days since they are involved in many physiologic and pathologic conditions, particularly cancer (67). Recent studies have highlighted the role of ncRNAs in regulating the expression of genes involved in ferroptosis in cancer (68). microRNAs (miRNAs) long non-coding RNAs (lncRNAs), and circular RNAs belong to ncRNAs whose roles in regulating gene expression have been frequently emphasized. Their role in regulating ferroptosis in lung cancer is discussed as below:

#### 2.4.1 miRNAs

miRNAs are a class of ncRNAs with 18-25 nucleotides long that can inhibit protein translation by affecting the expression of their target mRNAs (69). Tumor suppressor miRNAs are downregulated in cancers, and their targets are oncogenic proteins. miR-302a-3p is one of those downregulated tumor suppressor miRNAs that induces ferroptosis *via* targeting 3’-UTR of FPN mRNA in NSCLC. Ectopic induction of miR-302a-3p mimics provides a promising strategy against NSCLCs and sensitizes them to erastin, RSL3, cisplatin, and paclitaxel (70). Similarly, miR-324-3p expression is downregulated in cisplatin-resistant NSCLC cells, and its overexpression sensitizes resistant cells to cisplatin *via* targeting GPX4 (**Figure 1**) **(** 71). Moreover, tumor cells can release exosomes containing miRNAs to their surrounding microenvironment. miR-4443 is an oncogenic miRNA found abundantly in exosomes released from cisplatin-resistant NSCLCs and suppresses ferroptosis *in vivo* and *in vitro* through increasing FSP1 protein level indirectly by targeting methyltransferase-like 3 (METTL3), a molecule that causes N^6^-methyladenosine (*m* ^6^A) methylation of FSP1 (72) (**Figure 1**).

#### 2.4.2 lncRNAs

lncRNAs are a group of non-coding RNAs which are more than 200 nucleotides in length. Similar to miRNAs, these molecules affect gene expression through an extensive range of mechanisms. Moreover, they may act as sponges for miRNAs and inhibit their activity (73). For example, LSH can increase the expression of lncRNA-LINC00336 in lung cancer and thereby inhibit ferroptosis through sponging the activity of MIR6852, a miRNA that targets mRNA of a ferroptosis suppressor named cystathionine-β-synthase (CBS) (74) (**Figure 3**). Similarly, LSH is responsible for the downregulation of tumor suppressor lncRNA-P53RRA in NSCLC. P53RRA activates the p53 signaling pathway and induces ferroptosis *via* interacting with Ras GTPase-activating protein-binding protein 1 (G3BP1)(**Figure 3**) **(** 75). Furthermore, lncRNA-NEAT1 was shown to induce ferroptosis in NSCLC by increasing the expression levels of ACSL4 mRNA (76). Recently, Sui et al. showed that SAT1 mRNA could be stabilized by an RNA binding protein named U2AF2 and consequently lead to ferroptosis in LUAD. However, lncRNA-ASMTL‐AS1, which is responsible for recruiting U2AF2 and promoting SAT1 expression, is downregulated in LUAD cells, and thereby recovering its expression can inhibit malignant features of the cancer cells (77). Targeted delivery of ncRNAs can be considered a promising strategy against cancer. Gai et al. adopted a novel strategy to induce ferroptosis in NSCLC tumors by deploying folate-modified liposomes containing lncRNA-MT1DP combined with erastin. Since MT1DP increases the activity of miR-365a-3p, a miRNA that targets NRF2, subsequent deactivation of NRF2 (**Figure 2**) confers sensitivity to erastin-induced ferroptosis *in vivo* and *in vitro (* 78).

#### 2.4.3 circRNAs

circRNAs are more stable than miRNAs and lncRNA due to their covalently-closed structures, and their dysregulation is implicated in the progression of various cancer, including lung cancer (79). CircDTL is an upregulated oncogene circular RNA in NSCLC cells that inhibits ferroptosis *via* acting as a sponge for miR-1287-5p, a miRNA that targets GPX4. Moreover, inhibition of circDTL can increase the sensitivity of lung cancer cells to erastin *in vivo (* 80).

## 3 Ferroptosis and Prognosis of Patients With Lung Cancer

Up to now, thanks to The Cancer Genome Atlas (TCGA) and Gene Expression Omnibus (GEO) datasets, a significant number of studies have been conducted to analyze the expression profile of genes involved in ferroptosis in patients with lung cancer, particularly LUAD (81–93). As seen in **Table 1**, these studies have shown that *ferroptosis regulator genes* can predict the prognosis and overall survival of patients very efficiently. For example, various studies have shown that the expression of ALOX15, PEBP1, GLS2, and PHKG2 was associated with better prognosis and overall survival (low hazard ratio). In contrast, the expression of CISD1, ACSL3, FANCD2, SLC7A11, PGD, and GCLC was associated with poor prognosis (high hazard ratio). Therefore, the expression of these genes has the applicability of being used as biomarkers for predicting the prognosis of patients. Moreover, pathway analyses of ferroptosis-related genes have revealed that there is a close relationship between ferroptosis and immune system response, suggesting that future studies should pay more attention to this aspect of ferroptosis in lung cancer. Regarding the relationship between the immune system and ferroptosis, Huang et al. have shown that AKR1C1 negatively correlates with infiltrating level of immune cells, including CD4^+^ T cells, neutrophils, and dendritic cells in NSCLC. Moreover, its high-level expression negatively predicts overall survival and inhibits ferroptosis in NSCLC (98). Consistent with pathways discussed earlier, the involvement of signaling pathways including p53, fatty acid metabolism, ubiquitin-mediated proteolysis, and mTORC1 in ferroptosis is notable. In addition, several studies have evaluated the expression of ferroptosis-related long non-coding RNAs and their risk ratio (94–97). Descriptions and highlights of each conducted study are summarized in **Table 1**.

**Table 1:** Bioinformatic studies predicting prognosis of patients based on the expression of ferroptosis-related genes and non-coding RNAs.

Dataset used in the study	Low-risk	High-risk	Highlights of the study	Ref.
TCGA, GSE68465, and GSE72094	TLR4, PHKG2, PEBP1, GLS2, FLT3, and ALOX15	VDAC2, PGD, PANX1, KRAS, ALOX12B, ACSL3, CISD1, FANCD2, and SLC3A2	* “The expression of KRAS and PGD was positively related to tumor mutation burden, indicating that *KRAS and PGD* could serve as *novel biomarkers* for predicting *immunotherapy response rate*” * “VDAC2, GLS2, FLT3, TLR4, PGD, PANX1, PEBP1, ACSL3, CISD1, FANCD2, and SLC3A2 were of statistical significance” * “The four ferroptosis suppressor genes, ACSL3, CISD1, FANCD2, and SLC3A2, increased the *tumor’s stem cell-like* features and were all positively associated with *CD133 and CD44*” * “Ferroptosis process involves the *development of tumor immune evasion* (e.g., IL-17 signaling pathway).” * “*PEBP1* could be a promising treatment target and is positively related to *chemotherapy sensitivity*.”	(81)
TCGA and GSE68465	ALOX15, IL33, and GDF15	DDIT4 and HNF4A	* “Ferroptosis-related gene signatures can be used as a potential *predictor* for the prognosis of LUAD.” * “TCGA cohort showed *lower scores* in *immune-related cells, such as mast cells, neutrophils, dendritic cells (DCs), and T helper cells, with only natural killer (NK) cells showing higher scores*.” * “The *high-risk group* in the two cohorts *showed lower scores for type II and type I IFN responses*.”	(82)
TCGA, GSE72094, and GSE68465	NCOA4, GLS2, ALOX15, PEBP1, and PHKG2	ACSL3, PGD, ATP5G3, CISD1, and ALOX12B	* “The enriched gene sets in the *high-risk* group were mainly involved in pathways related to *glycolysis, mTORC1, MYC, G2/M checkpoint, unfolded protein response, E2F, hypoxia, mitotic spindle assembly, epithelial-mesenchymal transition, and late response to estrogen*.” * “Resting mast cells and resting dendritic cells can be identified as having a potential prognostic capacity in LUAD.” * “A total of 62.85% (308/490) of *autophagy-related genes* were found to be significantly correlated with risk scores.”	(83)
TCGA, GSE72094, and GPL15048	ANGPTL7, SLC1A4, GDF15, DUOX1, PHKG2, CDO1, LINC00472, DPP4, LINC00336, ALOX15, and GLS2	TXNRD1, DDIT4, SLC7A5, SLC2A1, RRM2, AURKA, ALOXE3, SLC7A11, and GCLC	* “The relationship between the ferroptosis-related genes and *tumor-infiltrating immune cells* was revealed by ANGPTL7 and M2 macrophages, ANGPTL7 and monocytes, GDF15 and M1 macrophages, LINC00472 and M2 macrophages, RRM2 and M1 macrophages, RRM2 and monocytes, and SLC2A1 and M1 macrophages.”	(84)
TCGA, GSE11969, GSE13213, GSE30219, GSE31210, and GSE41271	DUOX1, ALOX15, DPP4, CDO1, GDF15, and IL33	SLC7A11, GCLC, FANCD2, HELLS, ALOX12B, ALOXE3, TXNRD1, SRXN1, GPX2, DDIT4, SLC7A5, SLC2A1, RRM2, and AURKA	* “ALOX12B, ALOX15, GPX2, DDIT4, and GDF15 were increased and SLC2A1 and were decreased after erastin treatment.” * “ALOX15 was significantly low expressed in Ki67-high samples, while GPX2, DDIT4, and SLC2A1 were high expressed in Ki67-high samples.” * “Down-regulation of either GPX2 or DDIT4 could partially reverse the cell proliferation arrest.” * “Significantly enriched KEGG pathways include *cell cycle, complement, and coagulation cascades, p53 signaling, cellular senescence, and fatty acid metabolism*.”	(85)
TCGA, GSE72094, and GSE30219	AGER, ALOX15B, DPP4, GLS2, ISCU, PEBP1, PHKG2, SLC11A2,	ATP5MC3, CISD1, EGLN1, FANCD2, ITGA6, ITGB4, KRAS, NEDD4, SLC38A1, SLC7A5, STYK1, TFAP2A, VDAC1 AND VDAC2	* “Top five pathways enriched in the high-risk group were the *cell cycle, ubiquitin-mediated proteolysis, oocyte meiosis, homologous recombination and p53 signaling*.” * “The top five pathways enriched in the low-risk group were the *arachidonic acid metabolism, primary bile acid biosynthesis, alpha-linolenic acid metabolism, asthma, and intestinal immune network for IgA production pathways*.” * “Pathways of the *immune response* were significantly enriched in the 15-gene ferroptosis signature.”	(86)
TCGA	ALOX15, and PEBP1	ACSL4, GSS, ACSL3 and PGD	* “Gene’s mutation frequencies were higher in the high-risk group [*TP53* (53%), *TTN* (50%), *MUC16* (42%), *CSMD3* (40%), and *RYR2* (39%)].” * “The mainly enriched pathways included the neuroactive ligand-receptor interaction, metabolism of xenobiotics by cytochrome P450, steroid hormone biosynthesis, staphylococcus aureus infection pathway, IL-17 signaling pathway, retinol metabolic pathway.”	(87)
GSE68465, GSE41271, and GPL6884	CYBB and SAT2	CISD1, FADD and VDAC2	* “Several *immune-related pathways* were enriched in low-risk group, such as B cell receptor signaling pathway, T cell receptor signaling pathway, Intestinal immune network for IgA production, NOD line receptor signaling pathway, Fc epsilon Ri signaling pathway, Fc gamma R signaling pathway, and Graft *versus* host disease.” * “GSEA analysis showed the FRGS was highly associated with *immune status*. The enrichment score of aDCs, DCs, iDCs, pDCs, B cells, Macrophages, Mast cells, Neutrophils, T helper cells, Th1 cells, TIL and Treg was significantly increased in low-risk group. Meanwhile, low-risk group had a higher score of C–C chemokine receptor (CCR), the activity of checkpoint molecules, HLA, T cell co−stimulation and IFN Response Type II.”	(88)
TCGA GSE3141, GSE30219, and GSE31210	NOX1 and ALOX15	GSS, ACSL4, CISD1, SLC3A2, and FANCD2	* “Overall, the 12 top-ranked with highest mutations genes were shared between both sets KEAP1, NAV3, and FAT3, were expressed only in the high-risk group, while COL11A1, CSMD1, and ZNF536 were specifically expressed in the low-risk group.” * “The enrichment results revealed that processes related to poor survival in lung cancer patients, cancer microenvironment, immature B lymphocytes, early T lymphocytes and lung metastasis were significantly enriched in the high-risk group while processes related to COMP, lectin, TCRA, NOTCH1 target and hypoxia were significantly enriched in the low-risk group.” * “Ferroptosis-related risk score (FRRS) is involved in several *immune-signaling pathways*.” * “The gene expression levels of potential *immunotherapy targets, including CD276, PD-L1, and NKG2A, were significantly upregulated in the high-risk group*. Meanwhile, the expression levels of *VSIR and CD27 were significantly higher in the low-risk group than in the high-risk group*.” * “The *top three* genes that contributed most to FRRS were *CISD1, FANCD2 and SLC3A2*. The results illustrated that low CISD1 expression was significantly associated with favorable immunotherapeutic responses”	(89)
TCGA, GSE13213, and GSE72904	ALOX15, and DPP4	FANCD4, GCLC, and SLC7A11	* “Differentially expressed genes (DEGs) were mostly enriched in the ferroptosis pathway and immune-related pathways, such as human T-cell leukemia virus 1 (HTLV-1) infection pathway. These findings suggested that there exists crosslinking between ferroptosis and *tumor immunity* in NSCLC.” * “The GSE13213 dataset revealed differences in the scores of HLA class and type-I and -II immune interferon response.” * “The immune score of the subgroups in both TCGA cohort and the GSE13213 dataset was significantly different, especially the score of macrophages and mast cells.”	(90)
TCGA, GSE72094, and GSE68465	ARNTL, GLS2, HERPUD1, LPIN1, NCOA4, PEBP1, and TLR4	ACSL3, CISD1, DDIT4, EIF2S1, PANX1, RELA, RRM2, and YWAHE	* “ACSL3, YWHAE, DDIT4, PANX1, RELA, CISD1, EIF2S1, and RRM2 were overexpressed, while GLS2, PEBP1, ARNTL, NCOA4, LPIN1, HERPUD1, and TLR4 were downregulated in high-risk groups.” * “GAPDH, BIRC5, ERO1L, EIF2S1, SPHK1, ATIC, GNAI3, NAMPT, EIF4EBP1, and FADD are the top 10 autophagy-related genes that positively corrected with the risk score; 8/10 showed a significant elevated hazard ratio in LUAD.” * “ERN1, ATG16L2, CCR2, IKBKB, HSPB8, PRKCD, DAPK1, DRAM1, DLC1, and DAPK2 are the leading 10 that have negative relationships with the 15-gene signature risk score; three of them exhibited a decreased hazard ratio.” * “Enriched gene sets of HALLMARK collection in the high-risk group were mainly involved in pathways related to glycolysis, unfolded protein response, *mTORC1*, MYC, G2/M checkpoint, E2F, DNA repair, mitotic spindle assembly, ultraviolet radiation, hypoxia, cholesterol homeostasis, and reactive oxygen species, whereas the gene set concerned with metabolism of bile acids and salts was primary enriched in the low-risk group.”	(91)
TCGA	PEBP1, DPP4, ALOX15, GLS2, NCOA4 and PHKG2	ACSL3, GSS, PGD, FANCD2, SLC7A11, GCLC, CISD1, and ATP5MC3	* “PEBP1, ACSL3, NCOA4, PHKG2, and CISD1 were independent prognostic factors for overall survival.” * “Four kinds of immune cells showed higher infiltration levels in the high-risk group, including CD4 memory-activated T cells, M0 macrophages, M1 macrophages and activated dendritic cells, and three kinds of immune cells showed higher infiltration levels in the low-risk group, including resting mast cells, activated mast cells and eosinophils.” * “The results showed that the high-risk group had higher immune and stromal scores than those of the low-risk group.” * “PEBP1, CISD1 and NCOA4 were significantly down-regulated in the LUAD tissues.”	(92)
TCGA and GSE31210	ALOX15, DPP4, GLS2, PHKG2, and PEBP1	ATP5MC3, CISD1, FANCD2, GCLC, SLC7A11, ACSL3, ABCC1, and PGD	* “The higher risk group was significantly associated with *higher tumor stage, TP53 mutation, sex, and advanced tumor node metastasis (TNM) stage* in the TCGA cohort” * “Four immune‐related biological processes or molecular functions in KEGG were changed between the high‐ and low‐risk groups in the TCGA cohort, including the *intestinal immune network for IGA production, chemokine signaling pathway, TGF beta signaling pathway, and TOLL‐like receptor signaling pathway*” * “Four immune‐related biological processes or molecular functions in KEGG were changed between the high‐ and low‐risk groups in the TCGA cohort, including the *intestinal immune network for IGA production, chemokine signaling pathway, TGF beta signaling pathway, and TOLL‐like receptor signaling pathway*” * “Six immune‐related biological processes or molecular functions in GO were changed between the high‐ and low‐risk groups in the TCGA cohort, including somatic diversification of immune receptors, positive regulation of production of molecular, positive regulation of myeloid leukocyte cytokines, positive regulation of cytokine production, regulation of innate immune response, and activation of the innate immune response” * “The score of CD8+ T cells, iDCs, macrophages, mast cells, NK cells, Th1 cells, Th2 cells, Treg, antigen‐presenting cells (APC) coi-nhibition, cytolytic activity, HLA, inflammation‐promoting, MHC class I, para-inflammation, and T cell co-inhibition were significantly different between the low‐ and high‐risk groups in both TCGA”	(93)
TCGA	AC026355.1, AC124045.1, and AC025048.4	LINC01843, MIR193BHG, AC124045.1, AC091185.1, AC027031.2, ALO21707.2, ALO31667.3, and AL606834.1	* “lncRNA AL031667.3 increased with age, AC027031.2 was abundantly expressed in female patients, the expression of AC091185.1 and AC124045.1 was associated with TNM stage, that of AC091185.1, AC124045.1, AL021707.2, and LINC01843 was associated with pT stage, and that of AC124045.1, AL021707.2, AL031667.3, and MIR193BHG was associated with pN stage. Patients with decreased AC124045.1 expression were more likely to have distant metastases”	(94)
TCGA and GSE37745	CRNDE, AC106047.1, AC090559.1, AL691432.2, AC026355.1, AL034397.3, AC087752.3, VIM-AS1, HLA-DQB1-AS1, AC092171.5, LINCO0996, AC123595.1, ACO011477.2, and HSPC324	AL606489.1, LINC02081, AP000695.2, LINC01843, FAM83A-AS1, AP000695.1, and AC010980.2,	* “The Gene Ontology (GO) terms activation of innate immune response, innate immune response activating signal transduction, positive regulation of innate immune response, interleukin 1 mediated signaling pathway, and regulation of apoptotic signaling pathway were enriched in LUAD samples with high-risk scores. In contrast, CD8+ alpha beta T cell activation, T cell-mediated immunity, MAST cell-mediated immunity, regulation of leukocyte-mediated immunity, and regulation of lymphocyte-mediated immunity were enriched in LUAD samples with low-risk scores.” * “KEGG pathways were identified. Cell cycle, pancreatic cancer, *p53 signaling pathway*, pathogenic Escherichia coli, and *small cell lung cancer signaling pathways* were enriched in the high-risk group. Several immune response pathways, such as the intestinal immune network for IgA production, FC epsilon RI signaling pathway, autoimmune thyroid disease, allograft rejection, and graft *versus* host disease, were enriched in the low-risk group.”	(95)
TCGA, GSE3141, and GSE37745	ARHGEF26-AS1 C20orf197 MGC32805 LINC00324	LINC01116, LINC01137, and TMPO-AS1	* “The correlation expression between 7 lncRNAs and four most common ferroptosis-related mRNAs (FTH1, GPX4, ACSL4, PTGS2) verified the relationship between 7 lncRNAs and ferroptosis from another perspective.” * “Comparative analysis of immune cells and pathways confirmed the differences of HLA, MHC class I, para-inflammation, type I IFN response, type II IFN response, B cell, iDCs, mast cell, neutrophils, NK cell, T helper cell and TIL between two risk groups.”	(96)
TCGA, GSE30219, GES31210, and GSE31546	C5orf64, LINC01800, LINC00968, LINC01352, and PGM5-AS1	LINC02097, DEPDC1-AS1, WWC2-AS2, SATB2-AS1, LINC00628, LINC01537, and LMO7DN	* “The KEGG analysis results show that the 12 prognostic lncRNAs are mainly enriched in DNA replication pathway, B cell receptor signaling pathways, hematopoietic cell lineage pathway, and cell cycle pathway”	(97)

## 4 Treatments for Induction of Ferroptosis in Lung Cancer

Molecules such as system $X_{c}^{-}$ and GPX4 are potent inhibitors of the ferroptosis process, and two well-known small molecules which can inhibit them are erastin (99) and RSL3 (18), respectively. However, with a more detailed look, it can be concluded that the involvement of other signaling pathways might regulate key molecules of ferroptosis. For instance, Huang et al. have mentioned that treatment with erastin induces ROS production in NSCLC cells, which activate the p53 signaling pathway. Moreover, p53 can also inhibit the expression of SCL7A11 post-transcriptionally and subsequently induce ferroptosis (99). Recently, cisplatin (DDP) as a conventional chemotherapeutic agent has been shown to induce ferroptosis in different human cancer cell lines such as NSCLC by inhibiting GSH-GPX system activity, and its combination with erastin synergistically promoted treatment efficacy (100). Similarly, PRLX93936 is an analog of erastin, which its concurrent treatment with DDP can induce ferroptosis *via* GPX4 inhibition (101). Therefore, it seems that combining ferroptosis inhibitors with other treatments is an effective strategy to induce ferroptosis more potently. As seen in **Table 2**, the combination of erastin with other treatments has shown stunning anticancer properties through ferroptosis induction. In addition, the role of natural products in regulating ferroptosis has been highlighted in recent studies, suggesting them as future potential candidates in ferroptosis therapy, particularly in lung cancer. For better drug delivery, nanomaterials have received a lot of attention during recent years due to their ability for ferroptosis induction (117). Zero-valent-iron nanoparticle (ZVI-NP) is a type of nanomaterials conventionally used to remove pollutants from groundwater due to its high ability to produce ROS. Recently, their anticancer activity has been investigated *in vivo* and *in vitro* against lung cancer. Hsieh et al. have reported that silver-coated ZVI-NP (ZVI@Ag) can strongly induce lipid peroxidation and ferroptosis in lung cancer *via* GSK3β/β-TrCP-dependent degradation of NRF2. Moreover, it can provoke immune system activity by increasing cytotoxic CD8^+^ T cells and M1 (CD8^+^) anti-tumor macrophages (113). Another example of the applicability of nanoparticles for induction of ferroptosis in lung cancer is the synthesis of folate (FA)-modified liposome (FA-LP) nanoparticles containing erastin and lncRNA-MT1DP (E/M@FA-LPs), which has been mentioned earlier (78). Finally, another novel application of ferroptosis inhibitors such as erastin, IKE, RSL3 and sorafenib is administering them concurrent with radiotherapy to overcome radioresistance in NSCLC cells (116).

**Table 2:** Treatments for induction of ferroptosis in lung cancer.

Treatment	Cancer type	Target genes	Model	Description	Ref.
**Chemotherapy:**					
Erastin	NSCLC	↑p53/↓SCL7A11	*In vitro*	* “Erastin-induced ROS lead to the DNA damage response and stimulate p53 in A549 cells” * “Expression of p53 induced by erastin exposure contributes to the cytotoxic effect on A549 cells, leading to ferroptotic and apoptotic death.” * “p53 induced by erastin exposure exerts cytostatic effects on A549 cells”	(99)
Cisplatin	NSCLC	↓GSH-GPXs	*In vitro*	* “Cisplatin induced both ferroptosis and apoptosis in A549 cells” * “Silencing iron-responsive element binding protein 2 (IREB2) partially reversed the cytotoxicity of cisplatin, indicating the involvement of iron in cisplatin induced cell death” * “Additive effect observed in combination therapy of cisplatin and erastin”	(100)
Cisplatin (CDDP) with PRLX93936	NSCLC	↓GPX4	*In vitro*	* “Nrf2/Keap1 regulates sensitivity to RPLX93936/cisplatin in NSCLC cells.” * “Ferroptosis inhibitors and forced expression of GPX4 attenuated cell death caused by cisplatin and PRLX93936.”	(101)
Vorinostat with erastin	EGFR mutant LUAD	↓ xCT	*In vitro*	* “Vorinostat, a clinically used inhibitor targeting histone deacetylase, can robustly enhance the efficacy of ferroptosis inducers.” * “Cells with intrinsic or acquired resistance to EGFR-TKI display high sensitivity to ferroptosis inducers.”	(102)
Brusatol and erastin	NSCLC	↓ NRF2 ↑FOCAD-FAK	*In vitro*/*in vivo*	* “Treatment with NRF2 inhibitor, brusatol, increased the sensitivity of NSCLC cells to erastin-induced ferroptosis *in vitro* and *in vivo*, which depended on the upregulation of FOCAD partially” * “Brusatol can enhance the efficacy of chemotherapy *via* inhibiting NRF2 signaling pathway”	(34)
Erastin with acetaminophen (APAP)	NSCLC	↓NRF2/HO-1	*In vitro*/*in vivo*	* “Combination of erastin and APAP inhibited cell proliferation and induced ferroptosis” * Erastin and/OR APAP regulated intracellular ferrous iron * Erastin and/or APAP‐induced cell death *via* overgeneration of lipid peroxidation	(103)
Sorafenib combined with erastin	NSCLCs resistant to CDDP	↓ Nrf2/xCT	*In vitro*/*in vivo*	* “Sensitivity of NSCLC cells to CDDP is negatively associated with Nrf2 pathway activation” * “Erastin and sorafenib effectively induce ferroptosis in CDDP resistance cells by inhibiting the Nrf2/xCT pathway” * “Erastin/sorafenib restrains *in vivo* tumour growth in nude mice xenograft models”	(104)
Siramesine with lapatinib	LUAD	↓ HO-1	*In vitro*	* “Lapatinib and siramesine induce synergistic cell death in lung adenocarcinoma” * “Lapatinib and siramesine treatment increased reactive iron, ROS, and induced ferroptosis through decreasing heme oxygenase-1 (HO-1) protein expression.” * “Decrease in HO-1 expression was due to *proteasome degradation* and confirms that *Nrf2 is not implicated* in the regulation of HO-1”	(57)
Levobupivacaine (local anesthetic) and erastin	NSCLC	↑p53-↓SLC7A11-↓GPX4	*In vitro*/*in vivo*	* “Levobupivacaine inhibits proliferation and promotes apoptosis of NSCLC cells and represses invasion and migration of NSCLC cells.” * “Levobupivacaine induces ferroptosis of NSCLC cells”	(105)
Auranofin (AF, an antirheumatic drug)	NSCLC	↓TrxR ↓GPX4 ↑ HMOX1	*In vitro*/*in vivo*	* “p53 R273H cells were more vulnerable to AF-induced ferroptotic cell death due to downregulation of GPX4 and lipid peroxidation.” * “AF primes mutant p53 NSCLC cells for IL-15-stimulated NK cell mediated killing.” * “*Contrary*, it was observed that mutant p53 was no limiting factor in the activation of NRF2 and GSH levels, despite *reduced* expression of *SLC7A11* in the mutant p53 NCI–H1299 cells” * “To overcome the toxicity of AF-mediated TrxR inhibition, the data showed that all mutant p53 NSCLC cells *first* boosted their antioxidant defense capacities by *upregulation of pro-survival molecules, such as NRF2 and GSH*, to maintain their redox balance”	(106)
**Natural product therapy:**					
Artemisinin derivatives: Artesunate (ART) and Dihydroartemisinin (DHA)	NSCLC	↓VDAC and xCT ↑TFRC	*In vitro*	* “Artemisinin derivatives induce apoptosis and ferroptosis.” * “ROS is a key regulator of ART/DHA-induced apoptosis and ferroptosis” * “TFRC and VDAC were closely associated with the survival of lung cancer patients and can be used as potential therapeutic targets in lung cancer.”	(107)
Dihydroisotanshinone I (DT)	NSCLC	↓GPX4	*In vitro* and *in vivo*	* “DT inhibited the growth of lung cancer cells through apoptosis and ferroptosis and *in vivo* study inhibited metastasis of A549 cells in the nude mice model.”	(108)
Cryptotanshinone (CTN)	NSCLC	↓GPX4	*In vitro*	* “Cryptotanshinone induces ROS generation and caspase activity in lung cancer cell lines” * “CTN induces the lipid peroxidation iron-dependent” * “CTN induces apoptosis to the lower level than ferroptosis”	(109)
Curcumin	NSCLC	↑ACSL4 ↓ SLC7A11 ↓ GPX4	*In vivo* and *in vitro*	* ‘Curcumin inhibits tumor growth and promotes cells death *in vivo*” * “Curcumin suppresses cell proliferation and promotes cell death *in vitro*” * “Curcumin induces characteristic changes of ferroptosis in mice” * “Inhibition of autophagy attenuated curcumin-induced ferroptosis in A549 and H1299 cells”	(61)
Sulforaphane (SFN)	SCLC	↓SLC7A11	*in vitro*	* SFN inhibits growth and induces cell death in the SCLC cells * “SFN exhibits anticancer effects against SCLC cells *via* induction of ferroptosis” * “SFN-induced cell death was mediated *via* ferroptosis and inhibition of the mRNA and protein expression levels of SLC7A11”	(110)
6-Gingerol	Lung cancer	↓USP14 ↓GPX4 ↓ATF4 ↑NCOA4 and TfR1	*In vitro* and *in vivo*	* “6-Gingerol suppresses tumor growth and enhances the accumulation of ROS and iron.” * “6-Gingerol regulates the expression of autophagy and ferroptosis related proteins *in vivo* and *in vitro*.”	(60)
Ginkgetin with cisplatin(DDP)	NSCLC	↓NRF2/HO-1 axis ↓ GPX4 ↓ SLC7A11	*In vitro*/*in vivo*	* “Ginkgetin is synergized with DDP to increase cytotoxicity in NSCLC cells.” * “Ginkgetin disrupted redox hemostasis in DDP-treated cells, as demonstrated by the enhanced ROS formation and inactivation of the Nrf2/HO-1 axis.” * “Ginkgetin increased labile iron pool and lipid peroxidation and caused elevation of ROS formation, and apoptosis in DDP-treated NSCLC cells.”	(111)
Erianin	Lung cancer	↑Ca^2+^/CaM-dependent ferroptosis ↓SLC7A11	*In vitro*/*in vivo*	* “Erianin triggers cell death, inhibits cell proliferation, migration, and promotes cell cycle arrest in G2/M in lung cancer cells” * “Ferroptosis contributes to erianin-induced cell death in lung cancer cells” * “Erianin results in ferroptosis induction and exerts antitumor efficacy *in vivo*”	(64)
**Food additive for iron supplementation:**					
Ammonium Ferric Citrate (AFC)	NSCLC	↓GPX4-GSS/GSR-GGT axis	*In vitro*	* “Decreased the autophagy and cause elevated Fe2+ content and inducing oxidative stress injury consequently ferroptosis.” * “Inhibited the proliferation and invasion of NSCLC cell lines *in vitro*.” * “Promoted differential gene expression profiles of proliferation and autophagy.”	(112)
**Nanoparticle therapy:**					
ZVI-NP (Zero-valent Iron Nanoparticles)	Lung cancer	↑GSK3β/β-TrCP-dependent degradation of NRF2	*In vitro*/*in vivo*	* “Attenuated self-renewal ability of cancer and downregulated angiogenesis-related genes and caused lipid peroxidation, increased ROS, and ferroptosis.” * “Inhibited NRF2 activity and lung metastases *in vivo*.” * “ZVI-NP treatment promoted the M1 polarization induction-derived overexpression of *TNF-α*, while attenuated the expression of the M2 polarization gene *DC-SIGN*” * “ZVI-NP modulates immune cell profile in mouse model *in vivo*”.	(113)
Folate (FA)-modified liposome (FA-LP) enriched with erastin and MT1DP (E/M@FA-LPs)	NSCLCs	↓NRF2	*In vitro*/*in vivo*	* “E/M@FA-LPs sensitizes erastin-induced ferroptosis *in vitro*.” * “E/M@FA-LPs represses NRF2 levels to enhance oxidative stress.” * “E/M@FA-LPs could powerfully inhibit growth of subcutaneous xenografts.”	(78)
**Magnetic field therapy:**					
Magnetic field (MF) therapy concurrent with DDP and PTX treatments	Lung epithelial cancer cells (A549)	–	*In vitro*/*in vivo*	* “MF selectively inhibited malignant tumor cells” * “*Ferroptosis* was detected by co-incubation with ferrostatin-1” * “MF exposure led to ROS-dependent DNA damage and subsequent activation of DNA repair pathways” * “MF induced intracellular oxidative stress” * “MF sensitized tumor cells to conventional chemotherapy(DDP and PTX)”	(114)
**Radiation therapy:**					
Radiation combined with IKE and sorafenib	LUAD	↓ GPX4 ↓xCT	*In vitro*/*in vivo*	* “IKE and sorafenib, combined with stereotactic radiation therapy, suppress tumor growth in a mouse xenograft model of sarcoma and a patient-derived xenograft model of lung adenocarcinoma.” * “Radiation-induced cancer cell death is suppressed by ferroptosis inhibitors”	(115)
Radiation with erastin treatment	NSCLC	↓GPX4	*In vitro*	* “Erastin and IR exhibit a combined effect on killing cells” * “GPX4 expression is increased in the radioresistant cells and erastin inhibits GPX4 expression in the radioresistant cells” * “Knocking down GPX4 expression radiosensitizes NSCLCs cell to radiation in the radioresistant cell lines”	(116)

↓, Decreases the expression of; ↑, Increases the expression of.

## 5 Conclusion and Future Perspectives

This review provided an overall viewpoint and understanding of ferroptosis in lung cancer from molecular basis to prognostic and therapeutic significance. Although our understanding of ferroptosis is still insufficient, impressive efforts have been made during recent years to uncover the underlying mechanisms of ferroptosis, especially in NSCLC. To our knowledge, recent evidence has mainly focused on the role of system $X_{c}^{-}$ and GPX4 as main inhibitors of ferroptosis and other regulators and signaling pathways such as NRF2, p53, and UPS. These molecules have prominent roles in the ferroptosis process in lung cancer and are targeted by different treatments. Targeted delivery of drugs and ncRNAs involved in regulating ferroptosis and concurrent use of ferroptosis inhibitors alongside chemotherapy or radiotherapy have shown promising cytotoxic effects against lung cancer. Moreover, recent conducted bioinformatic analyses specifically address the impact of ferroptosis regulators in predicting patients’ overall survival and their close relationship with immune response. Therefore, it is suggested that future studies investigate the link between ferroptosis and immune response in lung cancer. As another limitation, the majority of these studies have focused on NSCLC, and there are very few studies that have evaluated ferroptosis in other subtypes of lung cancer (e.g., SCLC). These data together indicate that ferroptosis as a newly discovered cell death appears a promising target in lung cancer that can serve as a new candidate for its future treatment.

## Author Contributions

PT wrote the original draft. ZH drew the figures and their captions. SS assisted PT in writing the manuscript. SS and ZH both should be considered second authors. All authors contributed to the article and approved the submitted version.

## Conflict of Interest

The authors declare that the research was conducted in the absence of any commercial or financial relationships that could be construed as a potential conflict of interest.

## Publisher’s Note

All claims expressed in this article are solely those of the authors and do not necessarily represent those of their affiliated organizations, or those of the publisher, the editors and the reviewers. Any product that may be evaluated in this article, or claim that may be made by its manufacturer, is not guaranteed or endorsed by the publisher.
